# The Greek Versions of the HLS_19_ Health Literacy Instruments (HLS_19_-NAV-GR, HLS_19_-COM-GR, and HLS_19_-VAC-GR): Translation, Cultural Adaptation, and Descriptive Pilot Evaluation

**DOI:** 10.3390/healthcare13192541

**Published:** 2025-10-08

**Authors:** Angeliki Flokou, Panagiotis Theodorou, Dimitris A. Niakas, Petros Kostagiolas

**Affiliations:** 1 Department of Archives, Library Science and Museology, Ionian University, 49132 Corfu, Greece; theodorou.panagiotis@ac.eap.gr (P.T.); pkostagiolas@ionio.gr (P.K.); 2 School of Social Sciences, Hellenic Open University, 5 Evdoxou Str., 26331 Patra, Greece; dimitris.niakas@gmail.com; 3 Medical School, National and Kapodistrian University of Athens, 11527 Athens, Greece

**Keywords:** health literacy (HL), M-POHL HLS_19_, translation and cultural adaptation, Greece, HLS_19_-NAV-GR, HLS_19_-COM-GR, HLS_19_-VAC-GR, navigation HL, communicative HL, vaccination HL

## Abstract

**Background**: Health literacy (HL) is a key determinant of health outcomes and equity. The European Health Literacy Survey 2019 (HLS_19_) introduced three domain-specific instruments—HLS_19_-NAV, HLS_19_-COM-P-Q11, and HLS_19_-VAC. We present the translation, cultural adaptation, field testing, and descriptive pilot evaluation of their Greek versions (HLS_19_-NAV-GR, HLS_19_-COM-GR, HLS_19_-VAC-GR). **Methods**: Dual forward/back-translation and expert review (11 health professionals/academics) produced the final versions. A purposive, quota-guided field test (N = 71) approximated population distributions by sex, age, education, and geographical region. Test–retest stability (n = 16; ~12 days) was summarized primarily with intraclass correlation ICC (2,1), with Pearson/Spearman correlations reported secondarily. Internal consistency was assessed using ordinal alpha computed from polychoric (polytomous) and tetrachoric (dichotomous) correlations. We report item- and scale-level descriptive statistics for both the original polytomous (four-category, 1–4) responses and a dichotomous difficulty–ease scheme (1–2 vs. 3–4). Given the non-probability sampling in this pilot, the results are descriptive, not statistically representative. **Results**: Instruments were well accepted, requiring only minor revisions. Scales demonstrated high short-term stability and good internal consistency; inter-scale correlations were moderate, interpreted as associations among related but distinct constructs. Item distributions skewed toward Easy/Very Easy; several HLS_19_-VAC-GR items showed a clear ceiling, suggesting the need to consider harder items or a larger item pool in future validation. By scale, scores followed the descending order NAV, COM, and VAC. Distributions and ranking patterns broadly mirrored population-level findings from other countries. **Conclusions**: The adapted HLS_19_-NAV/COM/VAC-GR instruments are linguistically and culturally appropriate and prepared for large-scale validation, while items NAV9, COM4, and the VAC ceiling are flagged for further assessment.

## 1. Introduction

According to the World Health Organization (WHO), “health literacy represents the personal knowledge and competencies that accumulate through daily activities, social interactions, and across generations” [1]. At its core, HL comprises the ability to access, understand, appraise, and use information and services in ways that promote and maintain good health and well-being. It therefore goes beyond simply browsing websites, reading leaflets, or following recommended help-seeking routines. It involves exercising critical judgment of health information and the resources conveying it and engaging to articulate personal and societal needs for better health. When people have access to understandable and trustworthy information—and the skills to use it—health literacy enables informed decisions about personal health and active participation in collective health-promotion efforts that address the determinants of health [1]. At the service level, ensuring that such high-quality information is readily available across care settings is essential for effective communication and better decisions [2]. Higher satisfaction with the information patients receive is also associated with better disease control [3]. In contrast, evidence indicates that many adults, even those with higher education, struggle to navigate the healthcare system, make sense of health information, and work effectively with providers [4]. When health literacy is limited, it is associated with lower scores on decision-making outcomes [5], and overall outcomes worsen: hospitalizations and emergency use rise, preventive care falls, health status and mortality worsen, and costs increase; hospital length of stay is also longer among patients with low health literacy [6,7,8]. These burdens may fall disproportionately on vulnerable groups, including older adults and people with lower educational attainment [8]. Improving population health literacy—and making services easier to use for people with low health literacy—can help narrow inequities in outcomes [6].

At the policy level, the WHO’s Shanghai Declaration highlights the importance of health literacy in empowering citizens and enabling their engagement in collective health-promotion action within the 2030 Agenda for Sustainable Development [9]. Within Europe, Health 2020 positions health literacy as both a means and an outcome of efforts to empower people and enhance participation across communities and care. It calls for a whole-of-society approach—mobilizing multiple sectors and settings, and challenging health services to show leadership by making environments easier to navigate [10]. Against this backdrop, the first cross-national benchmark was the HLS-EU study (2009–2012) across eight countries, including Greece [11]. It introduced a comprehensive, multidimensional concept of health literacy, spanning healthcare, disease prevention, and health promotion, and operationalized it with the HLS-EU instruments (Q47 and short forms such as Q12). The findings highlighted wide cross-country variation and the need for harmonized measurement frameworks and broader HL definitions [12]. Following these recommendations, the WHO established the Action Network on Measuring Population and Organizational Health Literacy (M-POHL) in 2018 to support the availability of high-quality, internationally comparable data on population and organizational HL.

Under M-POHL, the multinational, standardized Health Literacy Population Survey 2019–2021 (HLS_19_) was coordinated across 17 countries in the WHO European Region to generate internationally comparable data [12,13]. HLS_19_ was built on the HLS-EU conceptual framework and instruments (Q47/Q16/Q12/Q6). It used the HLS_19_-Q12 as the general population measure [12,14] and, to reflect the field’s differentiation, offered four optional domain-specific modules: digital HL (HLS_19_-DIGI) [15,16], communicative HL with physicians in healthcare services (HLS_19_-COM) [17,18,19,20,21], navigational HL (HLS_19_-NAV) [22,23,24,25,26,27], and vaccination HL (HLS_19_-VAC) [15,28,29,30,31].

Despite the growing political and scientific interest in HL, Greece did not participate in the official international HLS_19_ survey. The present study addresses this gap by developing the first Greek versions of three specialized instruments—HLS_19_-NAV-GR, HLS_19_-COM-GR, and HLS_19_-VAC-GR—fully aligned with the official M-POHL adaptation protocol [13]. These instruments are intended to support national public health strategies and to enable comparable measurement across European and international contexts [12].

Specifically, this paper (i) documents the translation and cultural adaptation of HLS_19_-NAV-GR, HLS_19_-COM-GR, and HLS_19_-VAC-GR following internationally accepted guidelines for cross-cultural adaptation [13,32,33,34] and (ii) presents a descriptive pilot evaluation of score distributions, internal consistency, inter-scale relations, and short-term stability. Consistent with the pilot aim and sample size (N = 71), complete structural analyses (EFA/CFA, Rasch modeling) are reserved for a subsequent large-scale study. However, both polytomous and dichotomous scoring [12] are examined to assess comparability with international reporting and to explore potential effects of coding on reliability indicators.

## 2. Materials and Methods

### 2.1. Study Design, Permissions, and Instruments

After contacting the WHO Action Network on Measuring Population and Organizational Health Literacy (M-POHL) and obtaining permission to adapt the instruments into Greek, we initiated the translation and cultural adaptation of three Health Literacy Survey 2019 (HLS_19_) instruments for use in Greece: Navigational (HLS_19_-NAV) [26,27], Communicative Health Literacy with Physicians (HLS_19_-COM-P-Q11) [20,21], referred to hereafter simply as HLS_19_-COM, and Vaccination (HLS_19_-VAC) [30,31].

HLS_19_-NAV (12 items) assesses the ability to navigate the healthcare system at the macro (system), meso (organizational), and micro (interpersonal) levels, focusing on accessing, understanding, appraising, and applying information. HLS_19_-COM (11 items) evaluates communicative health literacy—patients’ communication and social skills when interacting with physicians. HLS_19_-VAC (4 items) captures the perceived ability to use vaccination-related information. All items in the three instruments begin with the same stem: “On a scale from very easy to very difficult, how easy would you say it is …” and are rated on a four-point polytomous Likert scale (1 = Very Difficult, 2 = Difficult, 3 = Easy, 4 = Very Easy), with higher values indicating greater HL. Overall scores were derived using two approaches: P-type, defined as the mean of item scores (1–4), which is then linearly transformed to a 0–100 scale, and D-type, defined as the percentage (0–100) of valid items with responses 3–4 (combined Easy/Very Easy). Using mathematical notation, the scoring rules can be expressed as follows (where k denotes the number of valid item responses per respondent):

(1) $$P-\mathrm{type}=\frac{100}{3k}\left(\sum_{i=1}^{k}c_{i}-k\right),\;\;\;\;\;\;\;c_{i}\in \{1,2,3,4\}$$

(2) $$D-\mathrm{type}=\frac{100}{k}\sum_{i=1}^{k}d_{i},\;\;\;\;\;\;\;\;\;\;\;\;\;\;\;\;\;d_{i}=\left\{\begin{array}{cc} 1, & c_{i}\in \{3,4\}\\ 0, & c_{i}\in \{1,2\} \end{array}\right.$$

In practice, the D-type scheme recodes the original polytomous responses into a dichotomous format by mapping {1,2} → 0 and {3,4} → 1, disregarding within-category gradations and thereby collapsing the four-category scale into a dichotomy along the difficulty–ease axis. Under this dichotomization, the D-type score is obtained by applying the P-type rule to the corresponding dichotomous responses *d_i_* ∈ {0, 1} —that is, taking the mean of the *d_i_* values and expressing it on a 0–100 scale. For the HLS_19_-NAV and HLS_19_-COM instruments, in either approach, the score is calculated only if at least 80% of the items have valid responses; otherwise, it is set to missing. For the HLS_19_-VAC, all four items must have valid responses for the score to be calculated. In all cases, a score of 0 indicates the lowest possible level, while 100 indicates the highest HL level. It is evident that the two scoring schemes result in discrete scores with different resolutions on the 0–100 scale: P-type scores can take 3*k* + 1 distinct values $\left(0,\;\frac{100}{3k},\;2\cdot \frac{100}{3k},\;\ldots ,\;3k\cdot \frac{100}{3k}=100\right)$ whereas D-type scores are restricted to only *k* + 1 $\left(0,\;\frac{100}{k},\;2\cdot \frac{100}{k},\;\ldots ,\;k\cdot \frac{100}{k}=100\right)$, where *k* is the number of scale items.

### 2.2. Instrument Adaptation (Translation—Cultural Adaptation—Expert Review)

The Greek adaptation followed the HLS_19_ Study Protocol, as proposed by M-POHL (The HLS_19_ Consortium of the WHO Action Network M-POHL, 2021) [13], aiming to ensure both linguistic accuracy and contextual relevance for the Greek population, while maintaining cross-country comparability of results.

Specifically, the process involved two consecutive stages: professional forward translation and backward translation. First, the forward translation from the original language, English, into Greek was carried out independently by two professional translators, following the recommended dual-panel method [32,33] and the HLS_19_ protocol; these were reconciled into a single, agreed-upon draft. Then, a third translator, blind to the originals and not involved in the forward phase, backtranslated the reconciled draft to check conceptual equivalence; discrepancies were discussed and resolved. Finally, all contributors validated the resulting intermediate Greek draft for each questionnaire. To strengthen quality assurance, the intermediate Greek versions of the HLS_19_-NAV, HLS_19_-COM, and HLS_19_-VAC were examined by an eleven-member expert panel (healthcare professionals and academics) who provided written comments, which were compiled into a summarizing table by the research team; all decisions were documented to maintain an audit trail.

### 2.3. Field Testing—Participants and Procedure

A field test was conducted in accordance with the procedure recommended by M-POHL, aimed at: (1) assessing whether participants understood the questions and response options, (2) estimating the average completion time, and (3) determining whether short, embedded clarifications were needed for potentially unclear terminology. The final sample was obtained via purposive, quota-guided, non-probability sampling to approximate the population distribution across the three M-POHL–recommended margins (age, gender, education level) and (our addition) geographic region. This led to a sample size of N = 71, exceeding the recommended pilot minimum (>30). To ensure geographic diversity, quotas were set based on the NUTS-1 (i.e., Nomenclature of Territorial Units for Statistics, first-level division representing the largest socio-economic areas) macro-regions used by Eurostat/ELSTAT: EL3 (Attica), EL4 (Aegean Islands & Crete), EL5 (Northern Greece), and EL6 (Central Greece).

The inclusion criteria used were adults aged 18 or older, residing in Greece for at least 12 months, possessing sufficient proficiency in the Greek language, and providing informed consent. Recruitment was conducted in waves, with ongoing monitoring of cell fills. Under-filled cells, such as those representing specific regions, were prioritized until the targets were reached.

All interviews were conducted via telephone by two trained members of the research team, who will also coordinate data collection in the upcoming full-scale survey [13].

### 2.4. Test–Retest Reliability Subsample

Short-term stability was assessed via test–retest reliability in a sample of 16 individuals who completed the instruments twice over an approximately 12-day interval under identical administration conditions. We summarized test–retest stability using the intraclass correlation coefficient ICC(2,1)—a two-way random-effects, absolute-agreement, single-measurement model. This model was chosen because the two occasions are treated as random, the focus is on absolute agreement (not just consistency), and decisions depend on individual scores. This is the standard approach for test–retest agreement when occasions or raters are not fixed. ICC values were interpreted using common standards: <0.50 = poor, 0.50–0.75 = moderate, 0.75–0.90 = good, >0.90 = excellent [35].

The approximately 12-day interval between administrations was chosen after baseline to reduce recall bias while minimizing the chance of actual change—an interval typically recommended for questionnaire reliability studies (~10–14 days; 2–14 days generally acceptable) and aligned with empirical practices in patient-reported outcomes (median 14 days; studies with strong ICCs, mean 12.88 days) [36,37].

Pearson’s r and Spearman’s ρ are reported as secondary measures for comparison, along with mean scores for test and retest, and their differences [test − retest]. For all estimates, we provided 95% bias-corrected and accelerated (BCa) bootstrap confidence intervals based on 10,000 resamples. The BCa method was used to account for potential bias and skewness in the sampling distributions.

### 2.5. Statistical Analysis

Data from the field test were used for a descriptive pilot evaluation. Internal-consistency reliability for each scale was examined under both data-handling types (polytomous, dichotomous) using the following indices [38]: First, Cronbach’s alpha for the full scale was computed, and the “alpha-if-item-deleted” coefficients were obtained to identify items whose removal would increase alpha (possible misfit). In parallel, ordinal alpha was reported, estimated from polychoric correlation matrices under polytomous handling and tetrachoric matrices under dichotomous handling, which are more appropriate for ordinal and dichotomous items, respectively [12,17,39]. Second, to complement alpha—which, in addition to reflecting the mean inter-item correlation, also depends on the number of items—inter-item correlations were summarized, and the mean inter-item correlation (MIC) was reported, interpreting MIC with commonly used benchmarks [33,40,41]. Third, corrected item–total correlations (ITC) were computed using polyserial coefficients under polytomous handling and point-biserial coefficients under dichotomous handling, to verify that each item was substantially and linearly related to the total score computed from the remaining items [40]. As a robustness/sensitivity check on the correlation metric, we also computed parallel Pearson inter-item, item–total, and alpha estimates for comparison with the commonly used Pearson-based acceptance thresholds.

Item-level percentage distributions for all three scales and both data types (polytomous/dichotomous) are reported in tables, accompanied by item microcharts (IMCs)—inline “chartlets” presented either as histograms (for polytomous data) or stacked bars (for dichotomous data). These relative frequencies are also visualized with stacked four-category percentage bars (1 = Very Difficult … 4 = Very Easy), ordered by the proportion of the combined Easy/Very Easy (3–4) categories, together with the Average Percentage Response Pattern (APRP) graph used by the HLS_19_ Consortium [13]—defined as the equal-weight mean of item-level response shares (based on valid responses) within each scale. As described by the HLS_19_ Consortium, APRP is a compositional summary that shows, for each response category, how often it is selected on average across an instrument’s items, facilitating quick overviews of response use and comparisons across populations and item sets. For item-level percentage calculations, denominators include only valid responses (1–4).

Scale scores for both scoring schemes (P-type/D-type) are summarized in tables with descriptive statistics, and their distributions are visualized with combo plots pairing normalized histograms and box plots. Histogram bin counts (for both scoring schemes) were set to reflect the discreteness of D-type scores —equal to the number of items (*k*) plus one— yielding 13 bins for NAV (*k* = 12), 12 for COM (*k* = 11), and 5 for VAC (*k* = 4). This choice facilitates comparability between the two scoring schemes and with published histograms. For scoring, DK/NA responses were treated as missing; computability thresholds followed the instrument-specific minimum-valid-response rules (Section 2.1)—i.e., no more than three items could be missing for NAV/COM and none for VAC. No imputation was performed.

Finally, pairwise inter-scale correlations as well as each scale’s correlation with the HLS_19_-Q12-GR scale (i.e., the three domain-specific HL scales versus the general HL scale) were reported as descriptive indicators of associations between related but distinct constructs [17]. HLS_19_-Q12-GR was administered using an existing Greek translation [42] developed by another team and scored according to the standard protocol to ensure comparability.

### 2.6. Ethics and Data Protection

The study complied with national data protection legislation and the EU General Data Protection Regulation (GDPR; Regulation (EU) 2016/679). Participation was voluntary, and all participants were informed about the study’s purpose and procedures; they were also informed that they could withdraw at any time, and verbal informed consent was obtained over the phone prior to data collection. Ethical approval was granted by the Research Ethics and Deontology Committee of the Ionian University (protocol codes [8813], [8814], [8815]; [27 May 2025]).

## 3. Results

### 3.1. Translation & Cultural Adaptation

Most issues concerned the HLS_19_-NAV instrument, for which six comprehension-related issues were documented: two for item NAV2 and one each for NAV3, NAV4, NAV8, and NAV12. Three of these were related to the Greek rendering of the verb “to judge.” The initial wording «να αξιολογήσετε» was replaced, based on field-test feedback, with «να κρίνετε» (NAV3, NAV8) and «να εκτιμήσετε» (NAV2); both are acceptable translations, but the replacement verbs were more readily understood by respondents in each item’s specific context.

For NAV2, although the expert panel’s recommendation to add clarifying examples was not implemented initially, the field-test findings confirmed it, and an explanatory parenthetical was added at the end of the item (e.g., general practitioner, specialist, hospital, health center). A further issue concerned the phrase “ongoing health care reforms” (NAV4): the initial Greek rendering «συνεχιζόμενες μεταρρυθμίσεις υγείας» was judged likely to require clarification of the term «συνεχιζόμενες»; the expert panel therefore recommended replacing it with «τρέχουσες μεταρρυθμίσεις υγείας» or «μεταρρυθμίσεις υγείας που βρίσκονται σε εξέλιξη» to improve comprehension.

One further adaptation concerned NAV12 (“to stand up for yourself if your health care does not meet your needs?”). The literal Greek rendering of “to stand up for yourself” («να υπερασπιστείτε τον εαυτό σας») was deemed not compatible with Greek usage in this context. The expert panel recommended a rights-based phrasing, «να υπερασπιστείτε τα δικαιώματά σας» (“to stand up for your rights”), to align with the institutional redress mechanisms that operate in practice. This wording is aligned with the national legal framework and the operation of the Office for the Protection of Patients’ Rights established in every hospital (Law 4368/2016, Art. 60) [43], which is responsible for providing information and support, receiving and processing complaints, and facilitating submissions to the Ombudsman and other oversight authorities.

Finally, for HLS_19_-COM and HLS_19_-VAC, the only modification concerned item VAC3, where the initial translation of “to judge” was—as in three HLS_19_-NAV items—replaced with the Greek verb «να κρίνετε».

### 3.2. Field Testing

The recorded completion times for respondents who participated in the field test were 3.9 min for HLS_19_-NAV (range: 2.0–9.0 min), 3.2 min for HLS_19_-COM (range: 2.0–8.0 min), and 1.1 min for HLS_19_-VAC (range: 0.5–3.0 min). In practical terms, completion required approximately 4 min for NAV (the longest), 3 min for COM, and 1 min for VAC (the quickest). The “Don’t know/Not applicable” (DK/NA) response option was selected only once (item COM11). However, it was observed that some formulations judged acceptable—with reservations—by the expert panel were not fully understood by the field-test participants and were therefore modified, as described earlier.

### 3.3. Test–Retest

Over a 12-day period, test–retest results for P-type scores demonstrated high temporal stability across all scales (Table 1). Agreement, summarized by the main measure—the intraclass correlation ICC (2,1)—ranged from 0.91 to 0.93. Pearson and Spearman correlations, reported as secondary measures, were also high, with values from 0.91 to 0.94 and 0.90 to 0.93, respectively. The corresponding 95% BCa CIs had sufficiently high lower bounds across all scales [44]. The mean test–retest change was small, and for each scale, the 95% BCa CI for the mean difference included zero, indicating that the difference was not credibly different from zero.

### 3.4. Reliability Analyses

Reliability analyses across all three instruments, HLS_19_-NAV-GR, HLS_19_-COM-GR, and HLS_19_-VAC-GR, showed high internal consistency, whether data were analyzed in their original polytomous form or transformed into a dichotomous format. As shown in Table 2, Cronbach’s alpha values were high (polytomous: 0.94, 0.91, 0.85; dichotomous: 0.89, 0.84, 0.79), while ordinal alphas—considered better suited for ordinal data—were even higher in all cases. Internal consistency was further supported by the “alpha-if-item-deleted” coefficients, which showed that each item positively contributed to its scale, as removing any one of them did not increase Cronbach’s or ordinal alpha compared to the full scale.

Additionally, the item-total correlations calculated using polyserial and point-biserial coefficients for polytomous and dichotomous data, respectively, consistently remained acceptable, indicating that each question contributed meaningfully to the construct being measured. Regarding item homogeneity as part of reliability, inter-item correlations, both pairwise and scale-averaged—computed using polychoric and tetrachoric coefficients for polytomous and dichotomous data, respectively—mostly fell within the commonly recommended range (>0.3) [40]. However, there was a notable exception with the NAV8-NAV9 pair correlation with dichotomous data, which was as low as 0.08, significantly lower than the next closest value. Apart from this, the NAV9 item consistently showed weaker associations, with the lowest coefficients in nearly all correlations it participated in, which were, however, within an acceptable range. Turning to the results for the HLS_19_-COM-GR scale, it is noteworthy that COM4 appeared in most of the lowest correlation coefficients (for polytomous data), while COM1 appeared as a pair peer both with COM4 in the lowest correlation (0.30) and, at the same time, with COM3 in the highest (0.82). Detailed item-level results are shown in Supplementary Tables S1–S3.

For completeness, Pearson-based reliability indicators were also calculated and are shown in Supplementary Tables S4–S6. These values were generally slightly lower than those obtained from polychoric/tetrachoric/polyserial estimation (which is expected due to the nature of the data). Their inclusion enables direct comparison with conventional interpretive thresholds/ranges, while the overall understanding of the reliability of the three instruments remains unchanged.

### 3.5. Item-Level Results

Frequency distributions were analyzed for each item across the three instruments and are summarized in Table 3, Table 4 and Table 5 in two formats: the original polytomous Likert (1–4) and the corresponding dichotomous (1–2 vs. 3–4). For the HLS_19_-NAV-GR scale (Table 3), responses in the Very Difficult (1) category were infrequent overall (mean 9.5%), ranging from 2.8% at NAV2 (“understanding forms”) to 23.9% at NAV12 (“finding out about quality”). The Difficult (2) category was the most frequent (mean 41.0%), ranging from 25.4% at NAV9 to 54.9% at NAV5, and was the modal response for 8 of 12 items, indicating that participants tended to view the tasks as difficult but mostly at a moderate rather than an extreme level. Easy (3) averaged 36.3% (22.5% at NAV4 to 52.1% at NAV2), while Very Easy (4) was less common (mean 13.3%; 9.9% at NAV4 to 19.7% at NAV8). However, when aggregated, the combined Very Difficult/Difficult (1–2) share averaged 50.5%, ranging from 32.4% at NAV9 to 67.6% at NAV4 (“understanding health care reforms”). The complementary combined Easy/Very Easy (3–4) share mirrored this pattern in reverse—from 32.4% at NAV4 to 67.6% at NAV9 (“getting an appointment”)—yielding an almost perfectly balanced distribution between difficulty and ease.

For the HLS_19_-COM-GR scale (Table 4), responses clustered strongly toward the Easy end of the scale. Responses in the Very Difficult category were rare (mean 2.7%), while responses in the Difficult category averaged 24.6%, peaking at COM4 (“receiving enough time in consultation”).

The Easy (3) category dominated (mean 51.9%), serving as the modal response in 10 of 11 items, with only COM4 shifting its mode to Difficult. Very Easy (4) averaged 20.8% but varied widely—from 8.5% at COM4 to over 40% at COM1 (“describing reasons for consultation”). Aggregated responses showed a clear tilt: the combined Easy/Very Easy (3–4) share exceeded 60% for all items except COM4 (40.9%), ranging from 62.0% (COM9) to 90.1% (COM1), with a mean of 72.7%, far outweighing the combined Very Difficult/Difficult (1–2) share (27.3% on average). Overall, COM items reflected relatively high perceived ease in communication with physicians, except for COM4, which differed substantially.

Similarly, the findings presented in Table 5 for the HLS_19_-VAC-GR scale show that respondents generally perceived vaccination-related tasks as easy; the Easy (3) category was modal across nearly all items, accounting for nearly half of responses (34.9–54.9, mean ~47%), while the combined Easy/Very Easy (3–4) share exceeded 70% for all items, ranging from 71.8% (VAC1, “finding information on recommended vaccinations for self/family”) to 85.9% (VAC2, “understanding why vaccinations may be needed for self/family”). This distribution pattern is consistent with a ceiling effect that restricts headroom at the upper end.

**Figure 1:**
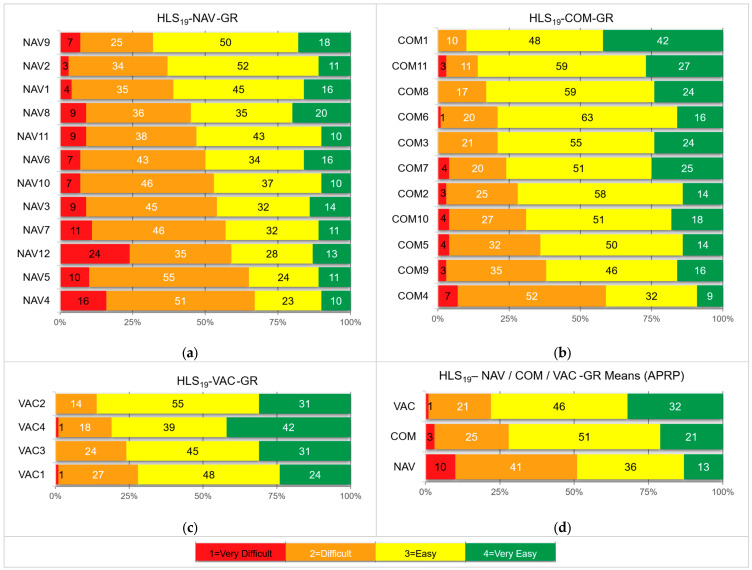
Stacked percentage bars by response category (1–4) for each item, with items ordered by the combined proportion of Easy/Very Easy (3–4). Panels: (**a**) HLS_19_-NAV-GR; (**b**) HLS_19_-COM-GR; (**c**) HLS_19_-VAC-GR; (**d**) scale-level means (APRP: Average Percentage Response Pattern—the equal-weight mean of item-level response shares within each scale, based on valid responses). Percentages are rounded; when necessary, the largest category for an item is adjusted so totals sum to 100%.

These item-level results are shown graphically in Figure 1. The first three panels display response profiles for each scale as stacked four-bar percentage graphs (1 = Very Difficult to 4 = Very Easy), ordered by the combined Easy/Very Easy (3–4) proportions in descending order. The graphs reveal apparent differences between scales: the combined Very Difficult/Difficult (1–2) categories versus the combined Easy/Very Easy (3–4) categories in the NAV scale show a nearly symmetric difficulty–ease profile centered around 50%. It begins at 32% difficulty for NAV9 (“getting an appointment”) and gradually increases to 68% for NAV4 (“understanding healthcare reforms”), with the intermediate items nearly linear between these extremes. Exactly half of the items fall below and half above the 50% line, creating a balanced pattern. In contrast, the COM scale is shifted toward lower overall difficulty: responses start with about 10% combined difficulty at COM1 (“describing reasons for consultation”) and rise in a nearly linear fashion, reaching 38% at COM9. However, this smooth gradient is sharply interrupted by the last item, COM4 (“receiving enough time in consultation”), where combined difficulty jumps to 59%. The VAC scale, by comparison, consistently shows low difficulty and high ease, with all four items clustering within a narrow range of aggregated difficulty levels (14–28%). In the bottom-right panel of Figure 1, these patterns are aggregated into scale-level means, represented as Average Percentage Response Pattern (APRP) graphs, ordered by the combined proportion of Easy/Very Easy responses (3–4) in descending order.

For orientation, the Spearman rank correlation between our Figure 1 item-difficulty rankings and those reported in other countries’ HLS_19_ studies [13] was *ρ* = 0.73 (HLS_19_-NAV-GR) and *ρ* = 0.76 (HLS_19_-COM-GR), reported descriptively.

### 3.6. Scale Level Results (P-Type and D-Type Scores)

Table 6 reports the total scores for the three instruments under both scoring methods. The distributions span the full or nearly full 0–100 range, and both means and medians show the same order: VAC → COM → NAV. As expected, D-type scores appeared more dispersed due to dichotomization, with median values shifted upward, which seems to reflect the higher prevalence of Easy (3) responses compared to Difficult (2). Shapiro–Wilk tests indicated non-normality in all distributions (all *p* < 0.05), with NAV being close to the significance threshold (*p* = 0.043). In the case of VAC, D-type scores showed a ceiling effect (skewness = –1.34).

Correlations with the overall HL scale (HLS_19_-Q12-GR) were moderate—r = 0.63 (NAV), 0.52 (COM), and 0.58 (VAC)—all within the discriminant validity range (0.40–0.70) [12] used as contextual benchmarks in full-scale validation studies. However, in this pilot study, we report these findings descriptively, interpreting them as associations consistent with related but distinct constructs (i.e., the three domain-specific scales are linked to overall HL but also measure different aspects), rather than as formal evidence of discriminant validity. Inter-scale correlations were also moderate (NAV–COM r = 0.64; NAV–VAC r = 0.47; COM–VAC r = 0.59), similarly indicating—descriptively—that the scales are connected but still represent separate constructs. Spearman’s coefficients (ρ = 0.61, 0.42, 0.55 with HLS_19_-Q12-GR; and inter-scale: NAV–COM ρ = 0.55, NAV–VAC ρ = 0.50, COM–VAC ρ = 0.57) matched the levels of corresponding Pearson values and led to the same conclusions.

For context, these findings are similar to those reported in the country-level results of the HLS_19_ project. The P-type correlations of NAV, COM, and VAC scales with HLS_19_-Q12-GR (0.63, 0.52, 0.58) closely resemble the international averages (~0.60, 0.52, 0.60). For D-type, NAV and VAC also align closely (0.55 and 0.50 vs. approximately 0.56 and 0.52), while COM was lower (0.33 vs. ~0.43), but remains very close to Austria (~0.34) [12].

**Figure 2:**
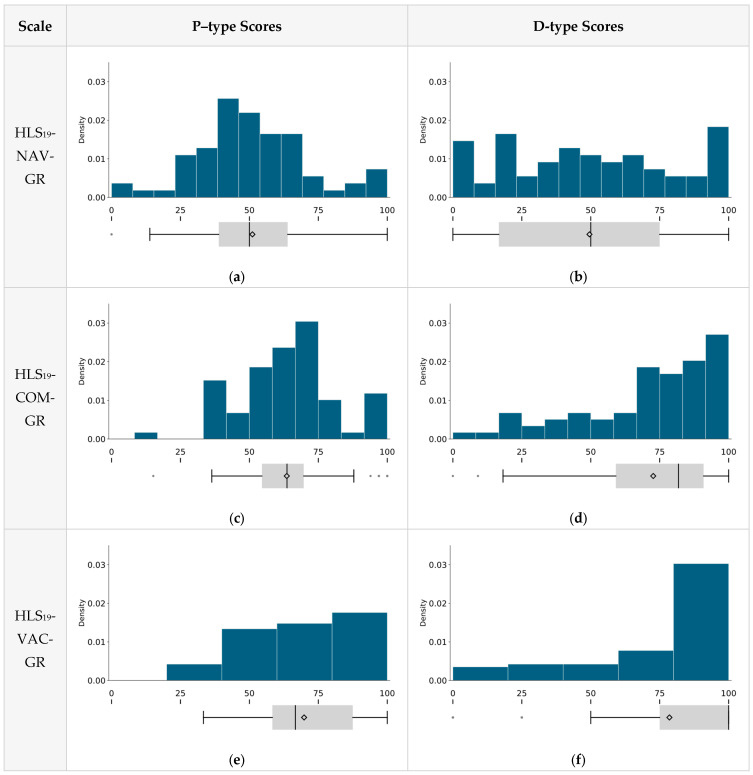
Distributions of P-type and D-type scores (0–100) for the Greek versions of the HLS_19_ instruments based on pilot data (N = 71). Each panel shows a normalized histogram and a box plot. Panels: (**a**) HLS_19_-NAV-GR P-type; (**b**) HLS_19_-NAV-GR D-type; (**c**) HLS_19_-COM-GR P-type; (**d**) HLS_19_-COM-GR D-type; (**e**) HLS_19_-VAC-GR P-type; (**f**) HLS_19_-VAC-GR D-type. P-type scores are the mean of item responses on the 4-point Likert scale, linearly rescaled to 0–100. D-type scores are the percentage (0–100) of items rated Easy or Very Easy (3–4) among valid responses. Histogram bins are set to the number of scale items plus one. In the box plots the line inside the box is the median, and the diamond indicates the mean.

Figure 2 shows the distributions. P-type scores appear tighter and more symmetric, while D-type scores are more spread out and skewed, with VAC exhibiting a clear ceiling effect. Again, for context, these shapes are qualitatively consistent with cross-country HLS_19_ histograms for COM (Q11/Q6), NAV, and VAC published in relevant studies [13,17,24].

## 4. Discussion

This field test and pilot evaluation, conducted as part of adapting three HLS_19_ instruments into Greek (HLS_19_-NAV-GR, HLS_19_-COM-GR, HLS_19_-VAC-GR), yielded a coherent psychometric profile and several practice-relevant observations.

### 4.1. Adaptation Issues and the Cultural Setting

Most of the adaptation work fell on NAV. The difficult part was the verb “to judge.” We changed the initial «να αξιολογήσετε» to «να κρίνετε» or «να εκτιμήσετε», depending on the context, which clarified the intent. NAV2 also needed a brief guide, so we added examples (“general practitioner, specialist, hospital, health center”) to anchor the service type. For NAV4, «συνεχιζόμενες μεταρρυθμίσεις υγείας» did not sound natural; «τρέχουσες μεταρρυθμίσεις υγείας» or «μεταρρυθμίσεις υγείας που βρίσκονται σε εξέλιξη» conveyed the same idea more plainly, and the latter was the finally chosen. NAV12 was rephrased from «να υπερασπιστείτε τον εαυτό σας» to the rights-based «να υπερασπιστείτε τα δικαιώματά σας», which is more natural in Greek usage and aligns with hospital patient-rights pathways and the national legal framework.

HLS_19_-COM required no adjustments, whereas HLS_19_-VAC required only one minor adjustment—«να κρίνετε» as the translation of “to judge” in VAC3.

These are just modest edits that, without changing the items’ structure, improve their clarity. Similar wording adjustments are noted in other HLS_19_ adaptations, especially regarding appraisal verbs and broad system terms like “reforms” [13,22]. The workflow was also shown to be important: first, an expert review, followed by a field test to confirm and identify necessary changes, providing a solid basis for the upcoming full-scale survey.

### 4.2. Internal Consistency and Item-Level Reliability

All three instruments showed high internal consistency under both data-handling schemes (polytomous/dichotomous), with Cronbach’s alpha and ordinal alpha well above the 0.70 benchmark [45]. Item–total correlations exceeded 0.30, and inter-item correlations were below 0.80, supporting an acceptable internal structure; “alpha if item deleted” analyses indicated no item that undermined reliability. Inter-item patterns offered insight, with the only concern being the lowest NAV inter-item correlation (0.08 for NAV8–NAV9). However, the corresponding item–total correlations were adequate, and Ordinal/Cronbach’s alpha did not increase when either item was removed, suggesting that these items tap different facets of navigation. More generally, the coexistence of a high scale-level (ordinal/Cronbach) α with some weak inter-item correlations indicates construct breadth rather than item redundancy; a scale can remain highly reliable when items cover different or complementary facets, as long as most items load meaningfully on the latent domain and the average covariance is adequate. Consistent with this, mean inter-item correlations (MICs) were high yet broader for NAV (polytomous 0.64, range 0.37–0.82; dichotomous 0.58, range 0.08–0.84) and COM (polytomous 0.58, range 0.30–0.82; dichotomous 0.55, range 0.21–0.83), whereas VAC showed a tighter, uniformly high pattern (polytomous 0.71, range 0.51–0.83; dichotomous 0.75, range 0.63–0.90) alongside the Easy/Very Easy ceiling—suggesting potential redundancy at the upper end. The national validation will quantify item targeting and information (e.g., Rasch) and, if necessary, consider minor wording refinements or harder items to improve upper-range discrimination. Nevertheless, given evidence from other countries that NAV9 may have weaker discrimination [24], a slight rephrasing might be necessary in the full-scale study to minimize interference from access or scheduling issues that are not directly related to health literacy. As a robustness check, we re-evaluated internal consistency using standard Pearson inter-item and item-total correlations; as expected, estimates were slightly lower but still supported the same conclusion of high reliability, showing robustness to the correlation measure.

### 4.3. Short-Term Stability

In terms of test–retest stability over an approximately 12-day period, the three scales examined using P-type (polytomous-based) scores showed high temporal stability. The agreement, summarized by ICC(2,1), ranged from 0.91 to 0.93 across the scales. Given the small retest sample (n = 16), these point estimates have limited precision, as reflected in wide 95% CIs; nevertheless, even in the worst case, CI lower bounds were above the ‘good’ threshold for every scale. Pearson *r* and Spearman *ρ* were similarly strong (*r* ≥ 0.91; *ρ* ≥ 0.90), with 95% CI lower bounds mainly in the “very good” range and once in the “good” range. However, we note cautiously that these correlations index association rather than agreement and may miss systematic shifts. Moreover, the mean test–retest change was small, and for each scale the 95% BCa CI for the mean difference included zero, indicating no evidence of a systematic shift between administrations. Taken together, the Greek adaptations show good short-term agreement with no detectable bias. However, final confirmation will be undertaken in the national validation.

### 4.4. Field Testing and Feasibility

Field testing showed that the adapted instruments were practical and easy to use, with short completion times (averaging 3.9, 3.2, and 1.1 min for NAV, COM, and VAC scales, respectively) and minimal missing responses. Only one “Don’t know/Not applicable” answer was recorded, indicating that the items were relevant to participants’ experiences. Only minor adjustments were needed after field testing, confirming the appropriateness of the final item wording. Of the few items requiring post-test modifications, most had already been identified by the expert panel, showing the real value of combining expert review with empirical testing during adaptation. Overall, our results suggest that the three adapted instruments are suitable for routine survey use in Greece.

### 4.5. Item-Level Results—APRP Patterns

In this pilot, item profiles appeared clear at face value. HLS_19_-NAV-GR showed a nearly balanced difficulty-ease pattern around the 50% line, with NAV4 being the most difficult and NAV9 the easiest. Item NAV9 “understand how to get an appointment with a particular health service” also showed the lowest mean inter-item correlations, indicating weaker overlap with certain navigation items; however, its corrected item–total relation was adequate, and ordinal α did not increase if deleted, consistent with facet breadth (e.g., scheduling/logistics) rather than item failure. This interpretation aligns with cross-country evidence from the HLS_19_-NAV validation in eight countries [24], where NAV9 was repeatedly flagged for comparatively weaker fit and occasional DIF, suggesting sensitivity to contextual/system factors beyond individual skill. In the national validation, NAV9 will be examined thoroughly using the same approach (Rasch fit and DIF checks). Depending on the results, any refinements will be made while aiming to maintain cross-country comparability where feasible.

HLS_19_-COM-GR was generally easier, but COM4 (receiving enough time in consultation) was noticeably more difficult than the other communication items. This mirrors the nine-country validation, where COM4 was often the hardest item and, in some datasets, showed weaker discrimination and indications of DIF [17]. In Greece, structural time constraints—short consultations and high patient throughput—likely limit dialogue and clarification, so responses to COM4 may reflect system-level factors in addition to communicative HL. This interpretation is consistent with local evidence on brief visits (≈10–15 min) and perceived time inadequacy (e.g., ~65% reporting insufficient time; only 24% of patients with ≥2 chronic conditions receiving >15 min consultations vs. the OECD PaRIS average of 47%) [46,47]. These contextual factors may help explain the higher share of Very Difficult/Difficult (1–2) responses on COM4. In the national validation, we will examine COM4 with Rasch fit diagnostics and DIF tests, implementing any warranted refinements.

HLS_19_-VAC-GR items clustered in high-ease categories, with fewer than one in four responses in the combined Very Difficult/Difficult (1–2) categories. The pronounced ceiling in several VAC items reduces responsiveness (true improvements may not register) and weakens discrimination among higher-literacy subgroups (effects attenuate at the top), limiting the instrument’s usefulness for detecting change and between-group differences at the upper end. In the national validation, we will assess upper range targeting and information (Rasch) and, if ceiling persists, consider wording adjustments and/or the inclusion of harder items. This pattern aligns with the HLS_19_ Consortium’s international report across eleven countries [13], which documented negatively skewed distributions with a marked ceiling and recommended developing and adding harder items (and expanding the item pool) to improve upper-range discrimination and sensitivity to change.

The APRP summaries indicated similar trends (more Easy/Very Easy in HLS_19_-COM-GR—and especially HLS_19_-VAC-GR—than in HLS_19_-NAV-GR). For context, these shapes resemble published results in the HLS_19_ Consortium’s international report [13]: the NAV extremes matched the corresponding published pattern (NAV9 lowest in the combined Very Difficult/Difficult categories; NAV4 highest), and the COM order aligned at the ends (COM1 easiest, COM4 hardest), though the COM4 difficulty in our data was higher. At the profile level, the Greek NAV APRP (10–41–36–13%) was very close to Belgium’s (12–40–38–11%), and the COM APRP (3–25–51–21%) resembled Germany’s (3–23–53–20%); VAC (1–21–46–32%) was similar to Norway’s (3–19–44–34%) and broadly to Austria’s (2–17–51–30%).

Beyond these profiles, the rankings of item difficulty also matched at the extremes and several intermediate positions: for NAV, the hardest item was NAV4, and the easiest was NAV9, in both our data and other studies; for COM, the hardest item was COM4, and the easiest was COM1, in both sources. Differences were modest in magnitude, and the informal rank-order correlation was high (*ρ* > 0.70). However, these cross-country anchors are provided for context only (similarities are noted but not interpreted, with formal comparison and modeling deferred to the main survey).

### 4.6. Scale-Level Results

At the scale level, mean and median scores followed the descending order VAC, COM, NAV for both scoring schemes, consistent with the decreasing ease ranking in Figure 1. The score distributions covered the full or nearly full 0–100 range. The shapes of their normalized histograms resemble those found in other HLS_19_-related studies [13,17,24]: smoother, mid-to-high unimodal P-type totals and stepwise, right-skewed D-type totals, especially for vaccination (indicating a ceiling effect tendency). As noted earlier, the four VAC items concentrate heavily in the top categories (3–4). This pattern carries over to the scale scores and is further amplified when moving from P-type to D-type scoring: intermediate gradations collapse at the threshold (1–2→0; 3–4→1), pushing the majority of scores above the midpoint upward and those below downward (Table S7). The result is a more polarized, stepwise, left-skewed distribution with reduced headroom at the upper end, further limiting the instrument’s discriminative ability among populations at the high end of vaccination literacy.

Regarding inter-scale associations, the three domain-specific HL scales (HLS_19-_NAV/COM/VAC-GR) showed moderate correlations (0.40–0.70) both among themselves and with the general HL scale (HLS_19_-Q12-GR). These values fall within ranges that full-scale studies often interpret as compatible with good discriminant validity; however, in this study, we treat them strictly as descriptive associations consistent with related but distinct constructs—no inference about discriminant validity is made from these correlations alone—and defer formal testing to the national validation—CFA (with AVE, Fornell–Larcker, HTMT) for convergent and discriminant validity, and multi-group CFA for measurement invariance across key subgroups.

### 4.7. Methodological Note

This study should primarily be viewed as a translation, cultural adaptation, and field-testing effort rather than a comprehensive psychometric validation. The pilot sample (N = 71) was intentionally designed to reflect the Greek population in terms of gender, age, educational level, and geographic region, in order to assess the comprehensibility, feasibility, and acceptability of the Greek versions of the HLS_19_-NAV, HLS_19_-COM, and HLS_19_-VAC instruments. The goal was not to produce inferential statistics but to confirm that the instruments work as intended in the Greek context, to identify items needing linguistic refinement, and to document initial descriptive data patterns.

Since the field test generated quantitative data, it was beneficial to provide a descriptive pilot evaluation of reliability indices, score distributions, and inter-item relationships. These analyses are not presented as proof of definitive validation but rather as a technical snapshot that shows whether the Greek instruments behave consistently with their theoretical expectations and international experience. In fact, some initial findings reflect observations from larger HLS_19_ studies—for example, NAV9 often showing weaker psychometric performance across countries, or COM4 standing out as unusually difficult compared to the generally smooth difficulty gradient of the HLS_19_-COM scale. Likewise, the ranking of item difficulties in the Greek sample largely matched those reported in multi-country studies, with only minor differences. Such similarities, even in a small pilot, offer reassurance that the Greek versions capture the intended constructs.

Therefore, the current study should be regarded as the essential first step: demonstrating that the instruments are linguistically and culturally appropriate, and providing a transparent preview of their preliminary statistical profile in Greece, while clearly postponing claims of validation to the large-scale survey. As highlighted in the literature, a well-conducted pilot study helps identify issues related to the effectiveness of the instruments and allows for the adaptation of both tools and the research design to the specific context, thereby enhancing the overall quality and relevance of the main study [48]. The full-scale validation work has already been prepared. It will be carried out with the upcoming national survey (*N* > 1000), excluding participants from the field test to ensure the independence of results, in accordance with international guidelines [49]. There, in addition to the basic analytical framework presented here, further robust psychometric methods (including CFA, Rasch modeling, and significance testing) will be applied, and the same descriptive tables and figures presented here will be replicated on the full dataset to enable direct comparison.

### 4.8. Limitations

As already stated, this was a non-probability (purposive, quota-guided) pilot with a small sample; therefore, the findings are descriptive and not statistically representative, with inferences restricted to feasibility and item-level performance signals. Population estimates and formal validation are deferred to the national study.

Telephone recruitment and quota completion may have introduced selection bias (e.g., differences in availability, coverage, or response propensity) that could shift item- and scale-score distributions. Telephone interviewing can also cause mode effects and social desirability bias. Recall and self-report biases might also affect responses.

## 5. Conclusions

This study developed Greek adaptations of the HLS_19_ domain-specific instruments—HLS_19_-NAV-GR, HLS_19_-COM-GR, and HLS_19_-VAC-GR—that are linguistically and culturally coherent and operationally feasible. In field testing, we observed high internal consistency and excellent short-term stability; inter-scale correlations were moderate and are interpreted descriptively as associations among related but distinct constructs. Item-level patterns highlight two priorities for national validation: HLS_19_-VAC-GR showed a clear ceiling (limited upper-end discrimination despite strong item–total relations), and COM4 (consultation time) was comparatively difficult, plausibly reflecting system-level time constraints. NAV9 showed lower pairwise overlap, consistent with a scheduling/logistics facet rather than item failure.

Given the non-probability, quota-guided design, and small test–retest sample, findings are descriptive and not statistically representative. The national validation will therefore (a) apply Rasch (PCM) to examine item/person targeting, information, threshold ordering, fit, and DIF; (b) assess convergent and discriminant validity with CFA and test measurement invariance; and (c) consider minor wording refinements or harder items (especially in VAC) only if warranted, while preserving cross-country comparability.

Practically, these instruments can support policy and program development in Greece—targeting navigation supports, informing clinician-communication initiatives, and guiding vaccination-literacy outreach—once validated at the national level. Furthermore, they can inform policies for lifelong learning programs that enhance healthcare professionals’ soft skills and digital abilities, promoting sustainable capacity-building across the system. They can also be used for benchmarking purposes by region and demographic groups, helping to identify disparities and monitor the impact of reforms as they are implemented. In any case, these tools will serve as valuable research resources and help fill a significant gap in the health literacy field for the Greek population.

## Figures and Tables

**Table 1:** Test–retest reliability over 12 days (n = 16), P-type scores (0–100).

Measure	HLS_19_-NAV-GR	HLS_19_-COM-GR	HLS_19_-VAC-GR
ICC (2,1)	0.91 [0.79, 0.97]	0.93 [0.86, 0.96]	0.91 [0.75, 0.97]
Pearson’s *r*	0.91 [0.80, 0.97]	0.94 [0.87, 0.98]	0.93 [0.76, 0.99]
Spearman’s *ρ*	0.90 [0.77, 0.95]	0.93 [0.83, 0.97]	0.90 [0.64, 0.99]
Mean (test)	47.74 [42.36, 53.13]	64.58 [60.04, 69.13]	57.29 [50.52, 64.06]
Mean (retest)	48.71 [42.80, 54.61]	65.72 [60.80, 70.64]	59.90 [52.08, 67.71]
Mean change (test–retest)	−0.96 [−3.65, 1.56]	−1.14 [−2.65, 0.38]	−2.60 [−5.21, 0.52]

Note: ICC (2,1) = two-way random-effects, absolute agreement, single-measurement intraclass correlation. Values are point estimates with 95% BCa bootstrap confidence intervals in brackets [L, U], based on 10,000 resamples. Negative values indicate higher retest scores.

**Table 2:** Reliability indicators for the HLS_19_-NAV-GR, HLS_19_-COM-GR, HLS_19_-VAC-GR instruments under polytomous and dichotomous response coding (N = 71).

Metric	Polytomous			Dichotomous		
	HLS_19_- NAV-GR	HLS_19_- COM-GR	HLS_19_- VAC-GR	HLS_19_- NAV-GR	HLS_19_- COM-GR	HLS_19_- VAC-GR
	(*k =* 12)	(*k =* 11)	(*k* = 4)	(*k =* 12)	(*k =* 11)	(*k =* 4)
Internal Consistency Reliability Coefficients						
Cronbach’s α, 95% CI [L, U]	0.94 [0.89, 0.96]	0.91 [0.85, 0.94]	0.85 [0.78, 0.90]	0.89 [0.84, 0.92]	0.84 [0.76, 0.89]	0.79 [0.66, 0.88]
Ordinal α, 95% CI [L, U]	0.95 [0.93, 0.97]	0.94 [0.90, 0.96]	0.91 [0.85, 0.94]	0.94 [0.91, 0.97]	0.93 [0.88, 0.96]	0.92 [0.83, 0.97]
Inter-item Correlations						
Mean	0.64	0.58	0.71	0.58	0.55	0.75
Min (pair)	0.37	0.30	0.51	0.08	0.21	0.63
	(NAV8–9)	(COM1–4)	(VAC1–4)	(NAV8–9)	(COM6–7)	(VAC1–4)
Max (pair)	0.82	0.82	0.83	0.84	0.83	0.90
	(NAV4–5)	(COM1–3)	(VAC2–3)	(NAV4–5)	(COM2–4)	(VAC2–3)
Corrected Item-total Correlations						
Mean	0.78	0.73	0.78	0.59	0.53	0.61
Min (item)	0.63	0.66	0.68	0.47	0.38	0.50
	(NAV9)	(COM6)	(VAC1)	(NAV4)	(COM7)	(VAC4)
Max (item)	0.89	0.85	0.89	0.75	0.69	0.72
	(NAV2)	(COM9)	(VAC3)	(NAV9)	(COM9)	(VAC3)

Note: Polytomous = original 4-point responses (1 = Very Difficult, 2 = Difficult, 3 = Easy, 4 = Very Easy); Dichotomous = 1–2 recoded to 0 (difficulty); 3–4 to 1 (ease). For polytomous coding, ordinal α and inter-item correlations used polychoric matrices; item–total correlations used polyserial coefficients. For dichotomous coding, ordinal α and inter-item correlations used tetrachoric matrices; item–total correlations used point-biserial coefficients. Values are point estimates with 95% bootstrap confidence intervals (CI) in brackets [L, U], based on 10,000 resamples. *k* denotes the number of items. Parenthetical labels identify the pairs/items contributing to the minima and maxima.

**Table 3:** Item-Level Distribution of HLS_19_-NAV-GR Responses in Polytomous and Dichotomous Formats with Item Microcharts (N = 71).

HLS_19_-NAV-GR Item	On a Scale from Very Easy to Very Difficult, How Easy Would You Say It Is …	Response Category (%)						IMC
		P-Type				D-Type		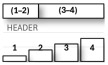
		1	2	3	4	(1–2)	(3–4)	
NAV1	… to understand information on how the health care system works? [e.g., which types of health services are available]	4.2	35.2	45.1	15.5	39.4	60.6	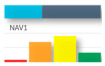
NAV2	… to judge which type of health service you need in case of a health problem?	**2.8**	33.8	**52.1**	11.3	36.6	63.4	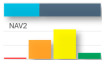
NAV3	… to judge to what extent your health insurance covers a particular health service? [e.g., are there any co-payments]	8.5	45.0	32.4	14.1	53.5	46.5	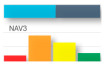
NAV4	… to understand information on ongoing health care reforms that might affect your health care?	15.5	52.1	**22.5**	**9.9**	**67.6**	**32.4**	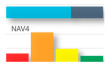
NAV5	… to find out about your rights as a patient or user of the health care system?	9.9	**54.9**	23.9	11.3	64.8	35.2	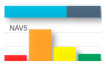
NAV6	… to decide for a particular health service? [e.g., choose from different hospitals]	7.0	43.7	33.8	15.5	50.7	49.3	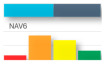
NAV7	… to find information on the quality of a particular health service?	11.3	45.0	32.4	11.3	56.3	43.7	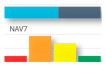
NAV8	… to judge if a particular health service will meet your expectations and wishes on health care?	8.5	36.6	35.2	**19.7**	45.1	54.9	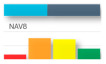
NAV9	… to understand how to get an appointment with a particular health service?	7.0	**25.4**	49.3	18.3	**32.4**	**67.6**	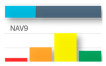
NAV10	… to find out about support options that may help you to orientate yourself in the health care system?	7.0	46.5	36.6	9.9	53.5	46.5	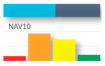
NAV11	… to locate the right contact person for your concern within a health care institution? [e.g., in a hospital]	8.5	38.0	43.6	9.9	46.5	53.5	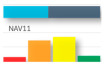
NAV12	… to stand up for yourself if your health care does not meet your needs?	**23.9**	35.2	28.2	12.7	59.1	40.9	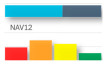

Note: Items are displayed in English, while responses pertain to the Greek-administered version (for source and permissions of use, see the “Acknowledgements” section at the end of the paper). Responses are shown in the polytomous 4-point format (1 = Very Difficult–4 = Very Easy) and the derived dichotomous format (1–2 = Combined Difficulty; 3–4 = Combined Ease). Percentages are shown to one decimal place; the largest category is adjusted so that totals sum to 100%. IMC = Item MicroChart: histograms (polytomous) and stacked bars (dichotomous). Bold percentages indicate column-wise minima and maxima.

**Table 4:** Item-Level Distribution of HLS_19_-COM-GR Responses in Polytomous and Dichotomous Formats with Item Microcharts (N *=* 71).

HLS_19_- COM-GR Item	On a Scale from Very Easy to Very Difficult, How Easy Would You Say It Is …	Response Category (%)						IMC
		P-Type				D-Type		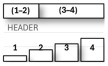
		1	2	3	4	(1–2)	(3–4)	
COM1	… to describe to your doctor your reasons for coming to the consultation?	**0.0**	**9.9**	47.8	**42.3**	**9.9**	**90.1**	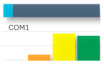
COM2	… to make your doctor listen to you without being interrupted?	2.8	25.4	57.7	14.1	28.2	71.8	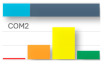
COM3	… to explain your health concerns to your doctor?	0.0	21.1	55.0	23.9	21.1	78.9	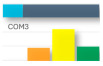
COM4	… to get enough time in the consultation with your doctor?	**7.0**	**52.1**	**32.4**	**8.5**	**59.1**	**40.9**	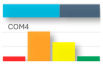
COM5	… to express your personal views and preferences to your doctor?	4.2	32.4	49.3	14.1	36.6	63.4	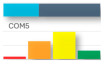
COM6	… to get the information you need from your doctor?	1.4	19.7	**63.4**	15.5	21.1	78.9	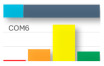
COM7	… to understand the words used by your doctor?	4.2	19.7	50.7	25.4	23.9	76.1	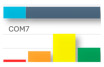
COM8	… to ask your doctor questions in the consultation?	0.0	16.9	59.2	23.9	16.9	83.1	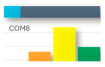
COM9	… to be involved in decisions about your health in dialogue with your doctor?	2.8	35.2	46.5	15.5	38.0	62.0	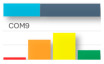
COM10	… to recall the information you get from your doctor?	4.2	26.8	50.7	18.3	31.0	69.0	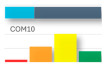
COM11 ^(^*^)^	… to use the information from your doctor to take care of your health?	2.9	11.4	58.6	27.1	14.3	85.7	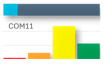

**Note**: Items are displayed in English, while responses pertain to the Greek-administered version (for source and permissions of use, see the “Acknowledgements” section at the end of the paper). Bold percentages indicate column-wise minima and maxima. For definitions and analytic details, see the Table 3 note. ^(^*^)^ N = 70 (COM11).

**Table 5:** Item-Level Distribution of HLS_19_-VAC-GR Responses in Polytomous and Dichotomous Formats with Item Microcharts (N = 71).

HLS_19_- VAC-GR Item	On a Scale from Very Easy to Very Difficult, How Easy Would You Say It Is …	Response Category (%)						IMC
		P-Type				D-Type		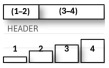
		1	2	3	4	(1–2)	(3–4)	
VAC1	… to find information on recommended vaccinations for you or your family?	1.4	**26.8**	47.9	**23.9**	**28.2**	**71.8**	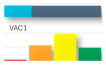
VAC2	… to understand why you or your family may need vaccinations?	0.0	**14.1**	**54.9**	31.0	**14.1**	**85.9**	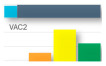
VAC3	… to judge which vaccinations you or your family may need?	**0.0**	23.9	45.1	31.0	23.9	76.1	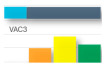
VAC4	… to decide if you should have a flu vaccination?	**1.4**	18.3	**39.4**	**40.9**	19.7	80.3	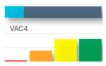

Note: Items are displayed in English, while responses pertain to the Greek-administered version (for source and permissions of use, see the “Acknowledgements” section at the end of the paper). Bold percentages indicate column-wise minima and maxima. For definitions and analytic details, see the Table 3 note.

**Table 6:** Descriptive Statistics of Total Scores for the three HLS_19_ instruments Based on Polytomous and Dichotomous Formats.

Statistic	P-Type Scoring (Polytomous)			D-Type Scoring (Dichotomous)		
	HLS_19_-NAV-GR	HLS_19_-COM-GR	HLS_19_-VAC-GR	HLS_19_-NAV-GR	HLS_19_-COM-GR	HLS_19_-VAC-GR
Mean	51.10	63.56	69.84	49.53	72.65	78.52
95% CI (Mean) Lower/Upper	46.11/59.06	59.48/67.64	65.00/74.67	41.77/57.29	66.32/78.98	70.88/86.16
Std. Deviation	21.08	17.24	20.43	32.79	26.75	32.26
Median	50.00	63.64	66.67	50.00	81.82	100.00
IQR	25.00	15.15	29.17	58.33	31.82	25.00
Range	100.00	84.85	66.67	100.00	100.00	100.00
Minimum/Maximum	0.00/100	15.15/100	33.33/100	0.00/100	0.00/100	0.00/100
Skewness/Kurtosis	0.32/0.65	0.14/0.42	−0.12/−0.94	0.09/−1.16	−1.00/0.07	−1.34/0.49
Shapiro–Wilk (*p*-value)	0.043	0.022	0.002	<0.001	<0.001	<0.001
Interscale correlations (Pearson’s *r*/Spearman’s *ρ)*						
HLS_19_-Q12-GR	0.63/0.61	0.52/0.42	0.58/0.55	0.55/0.56	0.33/0.36	0.50/0.47
HLS_19_-NAV-GR		0.64/0.55	0.47/0.50		0.48/0.46	0.42/0.46
HLS_19_-COM-GR			0.59/0.57			0.45/0.47

Note: P-type scores were calculated as the mean of item responses on the polytomous 4-point Likert scale, rescaled linearly to 0–100. D-type scores represent the percentage (0–100) of items rated as Easy or Very Easy (3–4) among valid responses. In both cases, higher values reflect higher HL.

## Data Availability

The raw data are available from the authors on reasonable request, subject to ethical and GDPR restrictions.

## Supplementary Materials

The following supporting information can be downloaded at: https://www.mdpi.com/article/10.3390/healthcare13192541/s1, Table S1. HLS_19_-NAV-GR reliability under polytomous and dichotomous coding: Ordinal α, α (if item deleted), inter-item r (mean/min/max + pair), and item–total r (mean/min/max) (N = 71); Table S2. HLS_19_-COM-GR reliability under polytomous and dichotomous coding: Ordinal α, α (if item deleted), inter-item r (mean/min/max + pair), and item–total r (mean/min/max) (N = 71); Table S3. HLS_19_-VAC-GR reliability under polytomous and dichotomous coding: Ordinal α, α (if item deleted), inter-item r (mean/min/max + pair), and item–total r (mean/min/max) (N = 71); Table S4. HLS_19_-NAV-GR reliability under polytomous and dichotomous coding: Cronbach’s α, α (if item deleted), inter-item r (mean/min/max + pair), and item–total r (mean/min/max) (N = 71); Table S5. HLS_19_-COM-GR reliability under polytomous and dichotomous coding: Cronbach’s α, α (if item deleted), inter-item r (mean/min/max + pair), and item–total r (mean/min/max) (N = 71); Table S6. HLS_19_-VAC-GR reliability under polytomous and dichotomous coding: Cronbach’s α, α (if item deleted), inter-item r (mean/min/max + pair), and item–total r (mean/min/max) (N = 71). Table S7. Theoretical score transitions from polytomous to dichotomous coding (P-type → D-type) for HLS_19_-VAC-GR (P-type: 4 items; 4^4^ = 256 response patterns).
