# Supplementary material for: The Greek Versions of the HLS19 Health Literacy Instruments (HLS19-NAV-GR, HLS19-COM-GR, and HLS19-VAC-GR): Translation, Cultural Adaptation, and Descriptive Pilot Evaluation
Source: Healthcare (Basel). 2025 Oct 8;13(19):2541. doi: 10.3390/healthcare13192541 (PMC12525262; doi:10.3390/healthcare13192541)
Supplement: Supplementary file 1 [file healthcare-13-02541-s001.zip › healthcare-3906891-supplementary.pdf]

Table S1. HLS_{19}-NAV-GR reliability under polytomous and dichotomous coding: Ordinal α, α (if item deleted), inter-item r (mean/min/max + pair), and item–total r (mean/min/max) ($N = 71$)

**HLS_{19}–NAV-GR Item**	**Ordinal alpha (If item deleted)**		**Inter-item Correlations**										**Corrected ITC**	
			**Mean**	**Min**	**(with)**	**Max**	**(with)**	**Mean**	**Min**	**(with)**	**Max**	**(with)**		
	P-type[1]	D-type[2]	P-type[3]					D-type[4]					P-type[5]	D-type[6]
NAV1	.95	.94	.67	.54	(NAV9)	.74	(NAV2)	.59	.50	(NAV12)	.75	(NAV10)	.81	.59
NAV2	.95	.93	**.73**	.62	(NAV12)	.81	(NAV5)	.67	.52	(NAV12)	.84	(NAV4)	**.89**	.66
NAV3	.95	.94	.61	.47	(NAV9)	.79	(NAV4)	.50	.23	(NAV7)	.76	(NAV4)	.74	.49
NAV4	.95	.93	.67	.46	(NAV6)	**.82**	**(NAV5)**	**.71**	.53	(NAV6)	**.84**	**(NAV5)**	.83	**.74**
NAV5	.95	.94	.69	.47	(NAV9)	**.82**	**(NAV4)**	.66	.35	(NAV9)	**.84**	**(NAV4)**	.86	.68
NAV6	.95	.94	.62	.46	(NAV4)	.81	(NAV2)	.54	.40	(NAV8)	.75	(NAV2)	.75	.54
NAV7	.95	.94	.63	.50	(NAV11)	.75	(NAV10)	.57	.23	(NAV3)	.80	(NAV10)	.77	.59
NAV8	.95	.94	.60	**.37**	**(NAV9)**	.73	(NAV1)	**.49**	**.08**	**(NAV9)**	.74	(NAV5)	.72	.48
NAV9	.95	.94	**.54**	**.37**	**(NAV8)**	.77	(NAV10)	.49	**.08**	**(NAV8)**	.78	(NAV10)	**.63**	**.47**
NAV10	.95	.93	.69	.58	(NAV8)	.77	(NAV9)	.66	.42	(NAV8)	.80	(NAV7)	.85	.69
NAV11	.95	.94	.60	.50	(NAV7)	.71	(NAV2)	.54	.38	(NAV3)	.77	(NAV4)	.72	.54
NAV12	.95	.94	.60	.43	(NAV9)	.73	(NAV5)	.57	.31	(NAV3)	.78	(NAV7)	.73	.59
Overall	.95	.94	.64	.37	(NAV8:9)	.82	(NAV4:5)	.58	.08	(NAV8:9)	.84	(NAV4:5)	.78	.59

Note: [1,3] Polytomous data–Polychoric correlations. [2,4] Dichotomous data–Tetrachoric correlations. [5] Polytomous data–Polyserial correlations. [6] Dichotomous data–Point-biserial correlations. Each row shows ordinal alpha if the listed item is deleted, the mean/min/max values of its inter-item correlations and its item–total correlation; peer items in column '(with)'; bold marks column extrema. The Overall row reports: (a) full-scale ordinal α; (b) for inter-item correlations, the global mean (MIC) and the global minimum/maximum with the corresponding item pair; and (c) for corrected item–total correlations, the mean of all ITCs.

Table S2. HLS$_{19}$-COM-GR reliability under polytomous and dichotomous coding: Ordinal α, α (if item deleted), inter-item r (mean/min/max + pair), and item–total r (mean/min/max) (N = 71)

**HLS$_{19}$–COM-GR Item**	**Ordinal alpha (If item deleted)**		**Inter-item Correlations**										**Corrected ITC**	
			**Mean**	**Min**	**(with)**	**Max**	**(with)**	**Mean**	**Min**	**(with)**	**Max**	**(with)**		
	P-type[1]	D-type[2]	P-type[3]					D-type[4]					P-type[5]	D-type[6]
COM1	.93	.93	.57	**.30**	**(COM4)**	**.82**	**(COM3)**	.53	.30	(COM11)	.82	(COM3)	.71	.45
COM2	.93	.92	.55	.36	(COM1)	.75	(COM4)	.61	.40	(COM7)	**.83**	**(COM4)**	.71	.64
COM3	.93	.92	.64	.51	(COM6)	**.82**	**(COM1)**	.59	.28	(COM6)	.82	(COM1)	.82	.57
COM4	.93	.93	**.52**	**.30**	**(COM1)**	.75	(COM2)	.52	.27	(COM6)	**.83**	**(COM2)**	.67	.46
COM5	.93	.93	.58	.45	(COM10)	.72	(COM9)	.51	.31	(COM10)	.76	(COM1)	.72	.48
COM6	.93	.93	.53	.36	(COM7)	.72	(COM11)	.46	**.21**	**(COM7)**	.75	(COM11)	**.66**	.43
COM7	.93	.93	.54	.36	(COM6)	.67	(COM10)	**.41**	**.21**	**(COM6)**	.59	(COM9)	.68	**.38**
COM8	.93	.92	.57	.44	(COM4)	.69	(COM11)	**.67**	.47	(COM7)	.82	(COM9)	.73	.64
COM9	.93	.92	**.67**	.54	(COM4)	.73	(COM11)	.67	.56	(COM4)	.82	(COM8)	**.85**	**.69**
COM10	.93	.93	.56	.36	(COM6)	.76	(COM1)	.55	.31	(COM5)	.70	(COM9)	.71	.53
COM11	.93	.92	.61	.46	(COM4)	.73	(COM9)	.56	.30	(COM1)	.75	(COM6)	.78	.51
Overall	.94	.93	.58	.30	(COM1:4)	.82	(COM1:3)	.55	.21	(COM6:7)	.83	(COM2:4)	.73	.53

Note: [1,3] Polytomous data–Polychoric correlations. [2,4] Dichotomous data–Tetrachoric correlations. [5] Polytomous data–Polyserial correlations. [6] Dichotomous data–Point-biserial correlations. Each row shows ordinal alpha if the listed item is deleted, the mean/min/max values of its inter-item correlations and its item–total correlation; peer items in column '(with)'; bold marks column extrema. The Overall row reports: (a) full-scale ordinal α; (b) for inter-item correlations, the global mean (MIC) and the global minimum/maximum with the corresponding item pair; and (c) for corrected item–total correlations, the mean of all ITCs.

Table S3. HLS$_{19}$-VAC-GR reliability under polytomous and dichotomous coding: Ordinal α, α (if item deleted), inter-item r (mean/min/max + pair), and item–total r (mean/min/max) (N = 71)

**HLS$_{19}$–VAC-GR Item**	**Ordinal alpha (if item deleted)**		**Inter-item Correlations**										**Corrected ITC**	
			**Mean**	**Min**	**(with)**	**Max**	**(with)**	**Mean**	**Min**	**(with)**	**Max**	**(with)**		
	P-type[1]	D-type[2]	P-type[3]					D-type[4]					P-type[5]	D-type[6]
VAC1	.91	.90	**.64**	**.51**	**(VAC4)**	.73	(VAC3)	.74	**.63**	**(VAC4)**	.83	(VAC3)	**.68**	.60
VAC2	.86	.89	.74	.68	(VAC1)	**.83**	(VAC3)	.77	.67	(VAC4)	**.90**	**(VAC3)**	.84	.62
VAC3	.84	.87	**.78**	.73	(VAC1)	**.83**	(VAC2)	**.81**	.71	(VAC4)	**.90**	**(VAC2)**	**.89**	**.72**
VAC4	.90	.94	.67	**.51**	**(VAC1)**	.79	(VAC3)	**.67**	.63	(VAC1)	.71	(VAC3)	.70	**.50**
Overall	.91	.92	.71	.51	(VAC1:4)	.83	(VAC2:3)	.75	.63	(VAC1:4)	.90	(VAC2:3)	.78	.61

Note: [1,3] Polytomous data–Polychoric correlations. [2,4] Dichotomous data–Tetrachoric correlations. [5] Polytomous data–Polyserial correlations. [6] Dichotomous data–Point-biserial correlations. Each row shows ordinal alpha if the listed item is deleted, the mean/min/max values of its inter-item correlations and its item–total correlation; peer items in column '(with)'; bold marks column extrema. The Overall row reports: (a) full-scale ordinal α; (b) for inter-item correlations, the global mean (MIC) and the global minimum/maximum with the corresponding item pair; and (c) for corrected item–total correlations, the mean of all ITCs.

Table S4. HLS19-NAV-GR reliability under polytomous and dichotomous coding: Cronbach's α, α (if item deleted), inter-item r (mean/min/max + pair), and item–total r (mean/min/max) (N = 71)

HLS19–NAV-GR Item	Cronbach alpha (If item deleted)		Inter-item Correlations										Corrected ITC	
			Mean	Min	(with)	Max	(with)	Mean	Min	(with)	Max	(with)		
	P-type[1]	D-type[2]	P-type[3]					D-type[4]					P-type[5]	D-type[6]
NAV1	.93	.88	.57	.46	(NAV9)	.64	(NAV10)	.39	.32	NAV12	.52	NAV10	.75	.59
NAV2	.93	.87	**.62**	.52	(NAV12)	.69	(NAV6)	.44	.33	NAV12	.53	NAV4	**.81**	.66
NAV3	.93	.88	.53	.41	(NAV9)	.70	(NAV4)	.33	.15	NAV7	.56	NAV4	.69	.49
NAV4	.93	.87	.59	.42	(NAV6)	**.73**	**(NAV5)**	.49	.34	NAV6	**.69**	**NAV5**	.77	.74
NAV5	.93	.87	.59	.39	(NAV9)	**.73**	**(NAV4)**	.45	.20	NAV9	**.69**	**NAV4**	.78	.68
NAV6	.93	.88	.54	.42	(NAV4)	.69	(NAV2)	.36	.27	NAV8	.52	NAV2	.70	.54
NAV7	.93	.88	.55	.43	(NAV11)	.68	(NAV10)	.39	.15	NAV3	.60	NAV10	.72	.59
NAV8	.93	.88	.52	**.31**	**(NAV9)**	.62	(NAV1)	.32	**.04**	**NAV9**	.49	NAV5	.67	.48
NAV9	.93	.88	**.46**	**.31**	**(NAV8)**	.66	(NAV10)	.32	**.04**	**NAV8**	.52	NAV10	**.58**	**.47**
NAV10	.93	.87	.60	.49	(NAV8)	.68	(NAV7)	.46	.28	NAV8	.60	NAV7	.78	**.69**
NAV11	.93	.88	.52	.43	(NAV8)	.61	(NAV4)	.37	.25	NAV3	.52	NAV4	.67	.54
NAV12	.93	.88	.53	.36	(NAV9)	.63	(NAV5)	.39	.20	NAV3	.60	NAV7	.68	.58
Overall	.94	.89	.55	.31	(NAV8:9	.73	(NAV4:5)	.39	.04	NAV8:9	.69	NAV4:5	.72	.59

Note: [1] Polytomous data–Cronbach α. [2] Dichotomous data–Cronbach α. [3] Polytomous data–Pearson correlations. [4] Dichotomous data–Pearson correlations. [5] Polytomous data–Pearson correlations. [6] Dichotomous data–Point-biserial correlations. Each row shows ordinal alpha if the listed item is deleted, the mean/min/max values of its inter-item correlations and its item–total correlation; peer items in column '(with)'; bold marks column extrema. The Overall row reports: (a) full-scale Cronbach α; (b) for inter-item correlations, the global mean (MIC) and the global minimum/maximum with the corresponding item pair; and (c) for corrected item–total correlations, the mean of all ITCs.

Table S5. HLS19-COM-GR reliability under polytomous and dichotomous coding: Cronbach's α, α (if item deleted), inter-item r (mean/min/max + pair), and item–total r (mean/min/max) ($N = 71$)

HLS19–COM-GR Item	Cronbach alpha (If item deleted)		Inter-item Correlations										Corrected ITC	
			Mean	Min	(with)	Max	(with)	Mean	Min	(with)	Max	(with)		
	P-type[1]	D-type[2]	P-type[3]					D-type[4]					P-type[5]	D-type[6]
COM1	.90	.84	.46	**.24**	**(COM4)**	**.66**	**(COM3)**	.29	.14	(COM11)	.52	(COM3)	.63	.45
COM2	.90	.82	.46	.28	(COM1)	.60	(COM4)	.39	.21	(COM1)	**.55**	**(COM8)**	.63	.64
COM3	.90	.83	.52	.40	(COM6)	**.66**	**(COM1)**	.36	.15	(COM6)	.52	(COM1)	.73	.56
COM4	.90	.84	.44	**.24**	**(COM1)**	.60	(COM2)	.29	.15	(COM6)	.52	(COM2)	.60	.46
COM5	.90	.83	.49	.39	(COM10)	.61	(COM9)	.31	.19	(COM10)	.44	(COM1)	.67	.49
COM6	.90	.84	**.43**	.28	(COM7)	.58	(COM5)	**.28**	**.11**	**(COM7)**	.48	(COM11)	**.59**	.43
COM7	.90	.84	.45	.28	(COM6)	.57	(COM10)	.24	**.11**	**(COM6)**	.38	(COM9)	.62	**.38**
COM8	.90	.82	.48	.38	(COM4)	.56	(COM11)	.41	.28	(COM7)	**.55**	**(COM2)**	.65	.65
COM9	.89	.81	**.56**	.47	(COM4)	.62	(COM11)	**.42**	.32	(COM1)	.54	(COM2)	**.78**	**.69**
COM10	.90	.83	.47	.29	(COM6)	.62	(COM1)	.34	.19	(COM4)	.48	(COM9)	.65	.53
COM11	.90	.83	.50	.39	(COM4)	.62	(COM9)	.32	.14	(COM1)	.48	(COM6)	.69	.51
Overall	.91	.84	.48	.24	(COM1:4)	.66	(COM1:3)	.33	.11	(COM6:7)	.55	(COM2:8)	.66	.53

Note: [1] Polytomous data–Cronbach α. [2] Dichotomous data–Cronbach α. [3] Polytomous data–Pearson correlations. [4] Dichotomous data–Pearson correlations. [5] Polytomous data–Pearson correlations. [6] Dichotomous data–Point-biserial correlations. Each row shows ordinal alpha if the listed item is deleted, the mean/min/max values of its inter-item correlations and its item–total correlation; peer items in column '(with)'; bold marks column extrema. The Overall row reports: (a) full-scale Cronbach α; (b) for inter-item correlations, the global mean (MIC) and the global minimum/maximum with the corresponding item pair; and (c) for corrected item–total correlations, the mean of all ITCs.

Table S6. HLS19-VAC-GR reliability under polytomous and dichotomous coding: Cronbach's α, α (if item deleted), inter-item r (mean/min/max + pair), and item–total r (mean/min/max) (N = 71)

**HLS19–VAC-GR Item**	**Cronbach alpha (if item deleted)**		**Inter-item Correlations**										**Corrected ITC**	
			**Mean**	**Min**	**(with)**	**Max**	**(with)**	**Mean**	**Min**	**(with)**	**Max**	**(with)**		
	P-type[1]	D-type2	P-type3					D-type4					P-type[5]	D-type[6]
VAC1	.84	.75	.55	**.43**	**(VAC4)**	.62	(VAC3)	.49	**.40**	**(VAC4)**	.60	(VAC3)	**.62**	.60
VAC2	.80	.74	.63	.57	(VAC4)	**.71**	**(VAC3)**	.50	.41	(VAC4)	**.63**	**(VAC3)**	.74	.62
VAC3	.77	.68	**.66**	.62	(VAC1)	**.71**	**(VAC2)**	**.57**	.47	(VAC4)	**.63**	**(VAC2)**	**.80**	**.72**
VAC4	.84	.79	.55	**.43**	**(VAC1)**	.66	(VAC3)	**.43**	**.40**	**(VAC1)**	.47	(VAC3)	.63	**.50**
Overall	.85	.79	.60	.43	(VAC1:4)	.71	(VAC2:3)	.50	.40	(VAC1:4)	.63	(VAC2:3)	.70	.61

Note: [1] Polytomous data–Cronbach α. [2] Dichotomous data–Cronbach α. [3] Polytomous data–Pearson correlations. [4] Dichotomous data–Pearson correlations. [5] Polytomous data–Pearson correlations. [6] Dichotomous data–Point-biserial correlations. Each row shows ordinal alpha if the listed item is deleted, the mean/min/max values of its inter-item correlations and its item–total correlation; peer items in column '(with)'; bold marks column extrema. The Overall row reports: (a) full-scale Cronbach α; (b) for inter-item correlations, the global mean (MIC) and the global minimum/maximum with the corresponding item pair; and (c) for corrected item–total correlations, the mean of all ITCs.

Table S7. Theoretical score transitions from polytomous to dichotomous coding (P-type → D-type) for HLS19-VAC-GR (P-type: 4 items; 4^4 = 256 response patterns).

P-type Score	D-type Score					Row total (number of patterns)
	0.0	25.0	50.0	75.0	100.0	
100.0					1	1
91.7					4	4
83.3				4	6	10
75.0				16	4	20
66.7			6	24	1	31
58.3			24	16		40
50.0		4	36	4		44
41.7		16	24			40
33.3	1	24	6			31
25.0	4	16				20
16.7	6	4				10
8.3	4					4
0.0	1					1
Column total (number of patterns)	16	64	96	64	16	256
Transition to Higher D-type Score	0	4	30	44	15	93
No transition (unchanged)	1	16	36	16	1	70
Transition to Lower D-type Score	15	44	30	4	0	93

Note: Cells display the number of theoretical response patterns, not sample frequencies. The table is derived from an exhaustive enumeration of the 4^4 = 256 possible patterns for HLS19-VAC-GR (four response categories per item, four items). P-type: mean of the 4-category responses (1–4) rescaled to 0–100 (e.g., 0, 8.3, 16.7, …, 100). D-type: dichotomization 1–2 = 0, 3–4 = 1, then average × 100 → five possible totals (0, 25, 50, 75, 100). The bottom three rows summarize how many patterns increase (green cells), remain unchanged (yellow cells), or decrease (orange cells) when mapped to the D-type total. The purpose is to illustrate information compression and the many-to-one mapping induced by dichotomization.
